# Effect of an Optimized Clinical Pathway Protocol Including Fascia Iliaca Compartment Block on Delirium and Postoperative Complications in Elderly Hip Fracture Patients

**DOI:** 10.3390/jcm14155284

**Published:** 2025-07-26

**Authors:** Carmen Corbella-Giménez, Elena Monge-Cid, Alba Gallo-Carrasco, Jorge Barros García-Imhof, Francisco Sánchez-Rodríguez, Jesús Díaz-García, Ignacio Vasserot, Maria José Anadon-Baselga, Matilde Zaballos

**Affiliations:** 1 Department of Anesthesiology and Reanimation, Hospital General Universitario Gregorio Marañón, 28007 Madrid, Spain; sonsaura@hotmail.com (C.C.-G.); emonge.hgugm@gmail.com (E.M.-C.); albagallo95@gmail.com (A.G.-C.); jbarrosgi@outlook.com (J.B.G.-I.); pacofran11@gmail.com (F.S.-R.); jdiazga1997@gmail.com (J.D.-G.); ivasserot@gmail.com (I.V.); 2 Department of Forensic Medicine, Psychiatry and Pathology, Faculty of Medicine Complutense University, 28040 Madrid, Spain; anadon@med.ucm.es

**Keywords:** hip fracture, delirium, optimized clinical pathway protocol, postoperative complications, fascia iliaca compartment block

## Abstract

**Background/Objectives**: Hip fractures are highly prevalent worldwide, primarily affecting frail elderly patients. Frailty increases the risk of complications like postoperative delirium, which negatively impacts outcomes, including morbidity and mortality. Current recommendations favor a multidisciplinary approach and effective pain control, often using preoperative peripheral nerve blocks. We aimed to evaluate a multimodal approach’s efficacy in reducing postoperative delirium and complications in geriatric hip fracture patients. **Methods**: This study was conducted between March 2020 and June 2022. A total of 144 patients evaluated prior to the implementation of an optimized clinical pathway protocol (OCPP) were compared to 117 patients evaluated following its implementation. The protocol included early preoperative evaluation, streamlined medication adjustments, prompt surgical intervention and fascia iliaca compartment block (FICB) for analgesia. In addition, early patient mobilization and resumption of oral intake were promoted. The primary outcome was the incidence of delirium during hospitalization. Secondary outcomes were a composite of 30-day mortality or major complications, duration of stay, hospital readmission after discharge and 1-year mortality. **Results**: The OCPP intervention significantly reduced the incidence of postoperative delirium from 44% to 29% (a 33% relative reduction; *p* = 0.017), the rate of major complications or death was 14.5% in OCPP group and 25.7% in the control group (*p* = 0.02). Significantly more patients in the OCPP group were mobilized within 24 h (74.4% vs. 41.3% in the control group, *p* < 0.001). The median time to ambulation was also shorter in the OCPP group: 65 h (IQR: 39–115) compared to 72 h (IQR: 48–119.75) in the control group (*p* = 0.028). No differences were observed on hospital stay and 1-year mortality. **Conclusions**: Among patients undergoing hip fracture repair the implementation of a OCPP significantly reduced the incidence of postoperative delirium and the rate of major complications or death. This improvement was associated with significantly earlier patient mobilization and ambulation. The OCPP was not associated with a lower hospital stay and lower rate of one-year mortality.

## 1. Introduction

Hip fracture is a prevalent condition, predominantly affecting frail elderly individuals. These patients often present with complex comorbidities, increasing their risk of postoperative complications and mortality. Notably, only 29% of patients undergoing hip fracture surgery regain their pre-fracture level of independence in activities of daily living within one year [1,2].

Delirium is among the most common complications reported in the perioperative period for these patients. It is a neuropsychiatric syndrome, defined as an organically induced disorder characterized by disturbances in consciousness and attention, alongside impairments in various cognitive functions including memory, orientation, thinking, language, and perception. Delirium has an acute onset and a fluctuating course, potentially lasting several days [3]. The reported incidence of delirium in this patient population ranges from 4% to 53% [4] and is associated with prolonged hospital stays, an increased risk of institutionalization, future cognitive dysfunction [4], increased mortality, and higher healthcare costs.

The etiology of delirium is multifactorial, arising from a complex interplay between patient-specific vulnerabilities (predisposing factors) and acute clinical stressors (precipitating factors) [3,4,5]. Key predisposing factors include advanced age, pre-existing cognitive impairment, and functional decline. The main precipitating events in the hospital setting are often the surgical trauma itself, uncontrolled pain, polypharmacy—especially the use of opioids and sedatives—and other physiological or environmental disturbances [3,4,5].

Currently, there is no definitive pharmacological treatment for delirium; however, appropriate perioperative management strategies can mitigate its incidence and severity [6]. Early and effective pain control, minimizing the use of opioids and benzodiazepines where feasible, is a cornerstone of improved patient care in this context. In this regard, regional anesthesia, particularly nerve blocks, plays a crucial role and should be considered in the absence of contraindications, irrespective of the primary anesthetic technique chosen for surgery [6].

While previous studies suggest that multifactorial strategies can be beneficial, their effect on broader clinical outcomes remains to be fully elucidated. Therefore, this study aimed to compare outcomes in hip fracture patients before and after the implementation of an optimized clinical pathway protocol (OCPP) incorporating the fascia iliaca compartment block (FICB). The primary goal was to assess for changes in the incidence of delirium as well as in a composite outcome of major postoperative complications and 30-day mortality.

## 2. Materials and Methods

The study was conducted according to the guidelines of the Declaration of Helsinki and approved by the Institutional Review Board of the Hospital General Universitario Gregorio Marañón (protocol code HIPF-20; approved 24 February 2021).

### 2.1. Study Population

To evaluate the associations of an optimized clinical pathway protocol in patients with hip fractures, we conducted a pre-post cohort study. The retrospective control group (pre-implementation) included patients with a hip fracture admitted from March 2020–March 2021 who received usual care. The prospective intervention group (post-implementation) included patients managed under the clinical pathway optimization protocol from January 2022–June 2022 Figure 1.

Patients aged ≥65 years old who were admitted for surgical treatment of hip fracture were eligible for inclusion.

### 2.2. Optimized Clinical Pathway Protocol

The preoperative management strategy incorporated the following key components:

**Anesthetic evaluation and surgical scheduling:** Prompt preoperative anesthetic evaluation was conducted, and surgical intervention was expedited.

**Geriatric optimization:** Priority was given to preoperative evaluation and optimization by geriatric specialists. This included a thorough assessment and management of anemia and nutritional status. The administration of psychoactive medications was avoided during the preoperative phase.

**Anticoagulation management:**

o In patients receiving warfarin, treatment was discontinued, and vitamin K was administered. Spinal anesthesia was allowed provided that the International Normalized Ratio (INR) was <1.5.
o **Direct oral anticoagulants (DOACs):**
  - For patients taking apixaban, edoxaban, or rivaroxaban, spinal anesthesia was permitted after a cessation period equivalent to at least two elimination half-lives of the respective drug.
  - For patients on dabigatran, a minimum withholding period of 48 h post-last dose was required before spinal anesthesia could be administered.
  - In individuals with renal insufficiency, these drug cessation intervals were adjusted according to the degree of renal impairment.

**Antiplatelet Therapy Management:**

o Antiplatelet medication was discontinued upon hospital admission except aspirin
o Surgical procedures were not delayed due to antiplatelet therapy cessation and the use of general anesthesia was encouraged.

**Analgesia:** The fascia iliaca block was performed promptly and repeated if surgery was delayed beyond 24 h. All blocks were conducted under ultrasound guidance using a high-frequency (13–6 MHz) linear transducer (SonoSite M Turbo; Bothell, WA, USA) and a 100 mm needle (Stimuplex, Braun, Melsungen, Germany). An in-plane, single-shot technique was employed to administer 30 mL of 0.375% ropivacaine combined with 8 mg of dexamethasone. Pain intensity was evaluated using the Numeric Rating Scale (NRS; an 11-point scale ranging from 0 [no pain] to 10 [worst imaginable pain]) and the Pain Assessment in Advanced Dementia (PAINAD) scale.

**Intraoperative management:** Routine intraoperative monitoring included electrocardiogram (ECG), pulse oximetry, and non-invasive blood pressure (NIBP). The choice between spinal or general anesthesia was made at the anesthesiologist’s discretion, adhering to current institutional practice. However, anesthesiologists were encouraged to:

o Avoid psychotropic drugs, opioids, anticholinergics, and antihistamines.
o Transfusion was considered when hemoglobin (Hb) levels fell to 8–9 g/dL. For patients with cardiac disease, a higher transfusion threshold of 9–10 g/dL was generally advised.
o Actively prevent hypothermia.
o Treat hypotensive events promptly.
o Furthermore, the protocol stipulated the administration of tranexamic acid and the avoidance of routine urinary catheterization.

**Postoperative management:** Postoperatively, patients were initially managed in the Post-Anesthesia Care Unit (PACU) before transfer to the ward upon clinical stabilization. The standardized analgesic regimen prioritized paracetamol, minimizing opioid and non-steroidal anti-Inflammatory drug (NSAID) administration. Early patient mobilization and resumption of oral intake were promoted. Management of anemia included transfusion therapy as previously mentioned.

### 2.3. Control Group

Patients cared according to the standard protocol of our hospital before the clinical pathway optimization protocol. For analgesia the patients received conventional standing and “as needed” parenteral or oral analgesics (opioids and acetaminophen) as determined by the treating physicians.

### 2.4. Data Collection and Outcomes

At the time of hospital admission, the following baseline data were recorded: age, sex, Body Mass Index (BMI), ASA physical status, comorbid conditions, pre-hospital living situation, functional status, presence of preexisting dementia or delirium, patient admission days, time elapsed until surgery, and type of fracture.

Intraoperative relevant data included: Characteristics of the surgical procedure and its duration; type of anesthesia administered, use of opioids and benzodiazepines, volume of intravenous fluids administered, red blood cell transfusions, incidence of intraoperative hypotension, requirement for vasopressor support, and administration of tranexamic acid.

Postoperatively, the following outcomes and data points were collected: pre-defined complications categorized as cardiovascular, respiratory, neurological, renal, wound infections or other surgical issues, total length of hospital stay, survival status and date of death.

### 2.5. Outcomes

The primary outcome was the incidence of delirium occurring at any point during hospitalization, assessed using the 4 A’s Test (4AT) delirium screening instrument [7,8]. Secondary outcomes included: a composite of 30-day mortality or major complications (defined as sepsis, pulmonary, and cardiovascular complications); requirement for blood transfusion; length of hospital stay; unplanned admission to the intensive care unit (ICU); hospital readmission following discharge; and 1-year all-cause mortality.

### 2.6. Statistical Analysis

Statistical analysis was performed using SPSS v29 (IBM Corp, Armonk, NY, USA). The normality of distribution for all continuous variables was evaluated using the one-sample Kolmogorov-Smirnov test. Normally distributed continuous data were presented as mean ± standard deviation (SD) and compared using the Student’s *t*-test. Non-normally distributed data were expressed as median with interquartile range [IQR] and compared using the Mann-Whitney U test. Categorical variables were presented as frequencies and percentages and compared using the chi-square or Fisher’s exact test, as appropriate. A two-tailed *p*-value < 0.05 was considered statistically significant.

### 2.7. Sample Size

The sample size was calculated to detect a clinically significant reduction in the primary outcome, the incidence of postoperative delirium. Based on a previous study reporting a delirium incidence of 34% with usual care, we determined that a reduction to 22% with our protocol would be a meaningful clinical improvement. To detect this difference (34% vs. 22%), with a statistical power of 80% (beta risk of 0.2) and a two-sided significance level (alpha) of 0.05, a minimum of 108 participants per group was required. It has been anticipated a drop-out rate of 10%.

### 2.8. AI-Assisted Manuscript Preparation

During the preparation of this manuscript, the authors used Google’s Gemini, a large language model, solely for the purpose of improving English language clarity and correcting grammar. All AI-generated suggestions were manually reviewed and edited by the authors to ensure the original meaning was preserved. The authors take full responsibility for the final content of this publication.

## 3. Results

**Figure 2:**
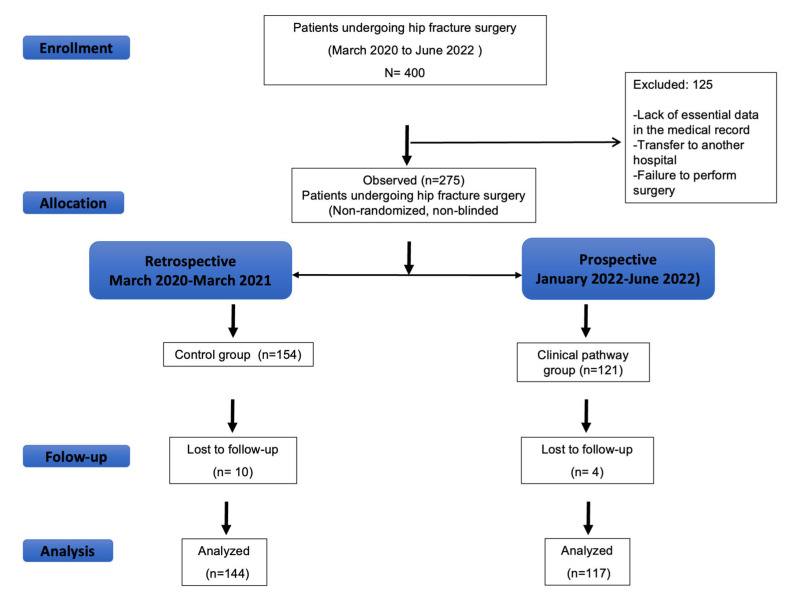
Study flow diagram.

Patients were enrolled from 1 March 2020 to 30 June 2022. Figure 2 shows the CONSORT diagram of the study. We analyzed 144 patients in the control group and 117 in the clinical pathway group.

Baseline characteristics were comparable between the two groups except for the type of fracture presented by the patients (Table 1).

There were no differences in the proportion of patients with preexisting dementia, degree of dependence and preoperative delirium at admission, however, there was a significant difference in the proportion of patients with high frailty index in the control group. (*p* = 0.008) Table 2.

Patients in the group of the clinical pathway had a significant shorter time to preoperative assessment and surgery including patients on antiplatelet or anticoagulants therapy Table 3.

Intraoperative management is reflected in Table 4. Patients in the clinical pathway group had less surgical drains, lower opioids and benzodiazepine administration and a higher proportion of PONV prophylaxis and tranexamic administration. Furthermore, patients in the clinical pathway group had less hypotension episodes and less intraoperative complications.

### Postoperative Management and Complications

The clinical pathway group demonstrated significantly improved postoperative outcomes compared to the control group. Specifically, the incidence of postoperative delirium was lower in the pathway group (29.3%; 95% CI, 21.0% to 38.0%) than in the control group (43.8%; 95% CI, 35.0% to 52.0%; *p* = 0.015), with an Odds Ratio of 0.52 (95% CI 0.31 to 0.98).

Given the higher proportion of frail patients in the control group at baseline, a specific subgroup analysis was conducted for participants with a frailty index > 4. In this high-risk population, the incidence of postoperative delirium remained significantly lower for patients in the optimized clinical pathway group (31%) versus the control group (47%), with an Odds Ratio of 0.51 (95% CI 0.26 to 0.98; *p* = 0.04).

Furthermore, the composite endpoint of major complications or death at 30 days was also significantly reduced (14.5%; 95% CI, 8.7% to 22.2% vs. 25.7%; 95% CI, 18.8% to 33.6%; *p* = 0.02).

There was a shorter time to food and drink intake and in ambulation in the clinical pathway group. No differences were observed on hospital stay and 1-year mortality. There were no other significant between-group differences between groups. Table 5.

## 4. Discussion

Our study’s principal finding indicates that the implementation of an optimized clinical pathway for hip fracture patients was significantly associated with a lower incidence of postoperative delirium. We also observed a reduced risk of major complications or death at 30 days in the post-implementation cohort. These findings suggest that a structured, multidisciplinary approach is a valuable strategy for improving key outcomes in this vulnerable population.

These results are consistent with previous multicomponent interventions that have reported reductions in delirium among hip fracture patients [10,11,12,13]. For instance, a study by Chuan et al. [10] found a similar 33% reduction in delirium incidence following a multidisciplinary intervention. Notably, unlike the findings of Chuan et al., our pathway was also associated with a significant reduction in the composite outcome of major complications or 30-day mortality.

The improved outcomes in our study are likely attributable to a combination of key interventions within our pathway. These include prompt preoperative anesthetic evaluation and expedited surgery, the strict avoidance of high-risk psychoactive medications, and prioritized geriatrician-led optimization. Furthermore, the effectiveness of our pathway is particularly noteworthy given the advanced age of our cohort, in which nearly 30% of patients were nonagenarians. Achieving these positive results in such a high-risk, frail population reinforces the substantial impact of a comprehensive, multidisciplinary-focused model of care.

Given the established link between pain control and improved outcomes in the elderly hip fracture population, the integration of peripheral nerve blocks, such as the fascia iliaca block, was a key component of our pathway. Supporting its utility, Mouzopoulos et al. demonstrated a significant reduction in delirium incidence among patients receiving the block (10.78%) compared to a control group (23.8%) [14]. While their reported incidence is lower than our findings, a notable discrepancy exists in the median age of the study populations. Their cohort had a median age of 72 years, substantially lower than the 86 years in our sample. This age gap likely accounts for the observed difference, given that advanced age is a principal risk factor for developing delirium.

Although the fascia iliaca block offers significant benefits for pain control and the mitigation of related complications, it is insufficient as a standalone intervention to prevent postoperative delirium [15,16,17,18,19]. Consequently, effective delirium mitigation likely requires a comprehensive, multidisciplinary strategy rather than a single-modality intervention [10,11,12,13]. Although not all optimization protocols have consistently demonstrated efficacy in reducing postoperative delirium, these discrepancies are likely attributable to variations in the strategies employed, the fidelity of their implementation, and differences in the study populations, which may present with varying comorbidity profiles [20].

Timely surgical intervention is a critical component in reducing mortality among patients with hip fractures. Evidence from a large cohort study by Vidan et al. (n = 2250) demonstrated that surgical delay is associated with increased mortality [21]. This risk, particularly for extensive delays exceeding 120 h, remained significant after multivariable adjustment for key confounders such as age, dementia, comorbidities, and pre-fracture functional status. In our cohort, despite the implementation of a clinical pathway, a significant proportion of patients did not undergo surgery within 48 h. This delay was primarily attributed to limited operating room availability rather than preoperative medical complications. Nevertheless, our clinical pathway successfully reduced the proportion of patients with surgical delays beyond 120 h from 16% in the control group to 7.7% in the intervention group (*p* = 0.043).

Similarly, in the anticoagulant-treated group, the clinical pathway significantly reduced the time to surgery, with 36% undergoing the procedure in < 48 h versus 3.5% in the control group (*p* = 0.019). This rapid management of anticoagulated patients is supported by recent evidence, as a systematic review has shown that proceeding with surgery under a defined protocol is safe and not associated with an increased 30-day mortality rate [22,23].

The mitigation of morbidity and mortality among hip fracture patients constitutes a formidable challenge in clinical practice. A study by Lee et al., employing a pre-post implementation design for a fascia iliaca block, observed a substantial decline in inpatient mortality from a baseline of 15% to 5.5% post-intervention [24]. Our findings indicate that the 30-day mortality in our clinical pathway group was analogous to the rate in Lee et al.’s post-intervention cohort. Strikingly, our control group demonstrated a 30-day mortality rate considerably lower than that of their pre-intervention baseline. The underlying reasons for this discrepancy remain speculative but could be rooted in a greater comorbidity burden within their patient sample. However, the lack of reported comorbidity data precludes a robust comparative analysis. Moreover, Lee et al. conceded that their results were likely confounded by the simultaneous introduction of other multidisciplinary interventions, obscuring the isolated effect of the block.

Reguant et al., in a non-randomized study, reported that a multidisciplinary intervention for hip fracture patients significantly reduced major complications and was associated with a protective effect on one-year mortality relative to a control group. Despite these benefits, no effect on the incidence of postoperative delirium was detected [25]. In our own cohort, we observed a similar reduction in the 30-day composite outcome of major complications or death favoring the intervention group. However, in contrast to the findings of Reguant et al., this short-term benefit did not translate into a significant difference in 12-month mortality between the groups.

### Limitations

Our study has several limitations that warrant discussion. First, the choice of the 4AT for delirium assessment may hinder direct comparability with studies employing different screening tools. Nevertheless, this was a pragmatic decision; the 4AT has robust validation for delirium assessment in surgical patients and its routine implementation at our center ensured consistent and reliable data collection by clinical staff [26].

Second, the retrospective design of the control group introduces a potential for selection bias. However, we believe this risk is substantially mitigated by the observed comparability of all key baseline characteristics between the groups, suggesting the cohorts were well-matched.

Finally, while the inherent heterogeneity of our patient cohort could be perceived as a limitation, we contend that it is a primary strength of the study. We intentionally adopted broad inclusion criteria, excluding no patients based on age or comorbidity. This pragmatic approach strengthens the study’s external validity, making our findings more directly generalizable to the complex, real-world patient populations encountered in routine clinical practice.

## 5. Conclusions

In conclusion, our findings indicate that an optimized clinical pathway is significantly associated with a reduction in postoperative delirium and major adverse events in patients with hip fractures. This evidence supports the adoption of structured, multidisciplinary pathways as a standard of care to improve outcomes for this vulnerable population.

## Figures and Tables

**Figure 1:**
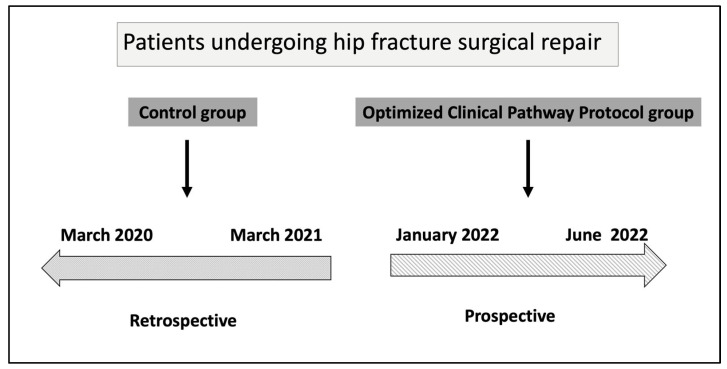
Time sequence of the study protocol.

**Table 1:** Baseline and procedural characteristics between control and clinical pathway groups.

	Clinical Pathway N = 117	Control N = 144	*p*
**Age (yr)**	83.40 ± 9.9; 86 [78.5–90.0]	84.2 ± 9.5; 86 [80.0–90.0]	0.43
**Older than 90 years; n (%)**	33 (28.2)	42 (29.2)	0.86
**Weight**	63 ± 13	62.6 ± 13.8	0.79
**Height**	159.1 ± 9.26	159.96 ± 8.65	0.49
**Body mass index (kg/m^2^).**	24.89 ± 4.56	24.63 ± 4.76	0.65
**ASA; n (%)**			
**I**	1 (0.9)	-	0.25
**II**	24 (20.5)	30 (20.8)	
**III**	84 (71.8)	95 (66)	
**IV**	8 (6.8)	19 (13.2)	
**Sex; n (%)**			
Female	83 (70.9)	105 (72.9)	0.72
Male	34 (29.1)	39 (27.1)	
**Comorbidity; n (%)**	116 (99.1)	138 (95.8)	0.1
**Origin; n (%)**			0.63
**Domicile**	100 (85.5)	120 (83.3)	
**Residence**	17 (14.5)	24 (16.7)	
**Diabetes; n (%)**	28 (23.9)	34 (23.6)	0.95
**Diabetes treatment; n (%)**			
**Oral antidiabetics**	16 (59.3)	12 (61.8)	0.12
**Insulin**	5 (18.5)	11 (32.4)	
**No treatment**	6 (22.2)	2 (5.9)	
**Glucose (g/dL)**	122 [101.0–142.0]	117 [98.0–144.0]	0.63
**Hemoglobin (mg/dL)**	12.5 [11.35–13.45]	12.7 [11.40–13.67]	0.59
**Hypertension; n (%)**	82 (70.1)	102 (70.8)	0.89
**Hypertension treatment; n (%)** (ACE-I or ARAII)	50 (57.5)	63 (48.1)	0.17
**Asthma/COPD; n (%)**	17 (14.5)	11 (7.6)	0.07
**OSAHS; n (%)**	5 (4.3)	2 (1.4)	0.15
**Active smoker; n (%)**	12 (10.4)	11 (7.9)	0.48
**Chronic kidney failure; n (%)**	17 (14.5)	17(11.8)	0.51
**Serum creatinine (mg/dL)**	0.87 [0.74–1.13]	0.8 [0.68–1.07]	0.075
**Estimated GFR; mL.min ^−1^.1.73 m^−2^**	47 [33.0–61.0]	50 [36.0–62.0]	0.26
**Liver disease**	6 (5.1)	6 (4.2)	0.71
**Coronary artery disease**	10 (8.5)	13 (9)	0.89
**Heart failure**	18 (15.4)	22 (15.3)	0.98
**Valvular heart disease**	16 (13.7)	18 (12.5)	0.77
**Atrial Fibrillation**	26 (22.2)	36 (25)	0.60
**Cerebrovascular disease**	14 (12)	17 (11.8)	0.96
**Urinary tract infection; n (%)**	2 (1.7)	5 (3.5)	0.38
**Antiplatelet therapy**	21 (17.9)	34 (23.6)	0.26
**Anticoagulation therapy**	30 (25.6)	38 (26.4)	0.89
**Chronic benzodiazepines treatment**	45 (38.5)	65 (45.1)	0.27
**Admission timing**			
From Monday to Thursday	80 (68.4)	79 (54.9)	
On Fridays	13 (11.1)	24 (16.7)	0.08
On weekends	24 (20.5)	41 (28.5)	
**Type hip fracture; n (%)**			
Subcapital femoral fracture	49 (41.9)	72 (50)	0.001
Pertrochanteric femoral fracture	47 (40.2)	70 (48.6)	
Basicervical femoral neck fracture	3 (2.6)	0	
Subtrochanteric femoral fracture	12 (10.3)	3 (1.4)	
Persubchanteric femoral fracture	5 (4.1)	0	
Other fractures	1 (0.9)	0	
**Type of surgical operation; n (%)**			
Hemiarthroplasty	48 (41)	66(43.8)	
Short intramedullary nail fixation	45 (38.5)	57 (39.6)	0.19
Long intramedullary nail fixation	21 (17.9)	15 10.4)	
Cannulated or compression screws	3 (2.6)	9 (6.3)	
Cemented implant (yes) *; n (%)	45 (93.75)	51 (77.27)	0.02
**Surgery duration (min)**	66.21 ± 28.77;	60.23 ± 24	0.07
	60 [46.5–83.5]	56 [45.0–70.0]	

ACE-I: Angiotensin-Converting Enzyme (ACE) inhibitors; ARA: Angiotensin II receptor antagonists; OSAHS: Obstructive sleep apnea/hypopnea syndrome. * Proportion in patients undergoing hemiarthroplasty. Values are number (proportion) or mean (SD) median (IQR [range]).

**Table 2:** Baseline characteristic of delirium status, dementia, cognitive impairment, dependency and frailty between control and clinical pathway groups.

	Clinical Pathway N = 117	Control N = 144	*p*
**Delirium status; n (%)**	18 (15.4)	27 (18.8)	0.47
**Dementia; n (%)**	39 (33.3)	64 (44.4)	0.11
GDS scale; n (%)			
1: No cognitive decline	77 (65.8)	81 (56.3)	0.40
2: Very mild cognitive decline	5 (4.3)	2 (1.4)	
3: Mild cognitive decline	9 (7.7)	15 (10.4)	
4: Moderate cognitive decline	5 (4.3)	7 (4.9)	
5: Moderately severe cognitive decline	10 (8.5)	16 (11.1)	
6: Severe cognitive decline	10 (8.5)	20 (13.9)	
7: Very severe cognitive decline	1 (0.9)	3 (2.1)	
**Barthel index; n (%)**			
**<20: total dependency**	6 (5.1)	13 (9)	0.61
**20–60: severe dependency**	26 (22.2)	33 (22.9)	
**61–90: moderate dependency**	41 (35)	51 (35.4)	
**>90: mild dependency**	44 (37.6)	47 (32.6)	
Clinical Frailty Scale *; n (%)			
1: very fit	7 (6)	10 (6.9)	
2: Well	20 (17.1)	24 (16.7)	0.008
3: Well, with treated comorbid disease	23 (19.7)	19 (13.2)	
4: Apparently vulnerable	25 (21.4)	21 (14.6)	
5: Mildly frail	23 (19.7)	16 (11.1)	
6: Moderately frail	17 (14.5)	45 (32.6)	
7: Severely frail	2 (1.7)	9 (6.5)	

GDS: Global Deterioration Scale * Rockwood et al. [9]. Values are number (proportion).

**Table 3:** Perioperative management.

	Clinical Pathway N = 117	Control N = 144	Effect Size (95%CI)	*p*
**Time to preoperative assessment (hours)**	32.86 ± 28.97; 21 [14.0–44.0]	49.14 ± 56.15; 36 [19.0–62.0]	–8.56 [–15 to –3]	0.002
**Time to surgery (days)**	2.68 ± 1.87; 2 [2.0, 3.0]	3.44± 2.44; 3 [2.0–4.0]	–1 [–1 to 0]	0.001
**Time to surgery (hours)**	66.50 ± 45.49; 52.46 [41.75–76.50]	79.23 ± 55.41; 69 [44.0–99.75]	-–9.5 [–19.5 to –1.52]	0.02
**Surgery within 48 h**	49 (41.9)	43 (29.9)	1.69 [1.01 to 2.82]	0.043
**Time to surgery in patients on antiplatelet or anticoagulants therapy (days)**	2.76 ± 1.42; 2.5 [2.0–4.0]	4.03 ± 2,8; 3 [2.0–5.0]	–1 [–1 to 0]	0.006
**Time to surgery in patients on antiplatelet or anticoagulants therapy (hours)**	66.22 ± 33.8; 59.5 [41.87–87.51]	90.86 ± 62.12; 76 [49.0–114.75]	–16.41 [–29 to –2.45]	0.016
**Time to surgery in** **patients on anticoagulants (days)**	2.77 ± 1.17; 2 [2.0–4.0]	4.73 ± 3.23; 4 [3.0–5.5]	–1 [–2 to –1]	0.002
**Time to surgery in** **patients on anticoagulants (hours)**	67.42 ± 30.35; 58 [41.5–98.0]	106.13 ± 68.71; 92 [66.0–122.0]	–26 [–47 to –9.49]	0.005
**Preoperative complications**	20 (17.1)	47 (32.6)	0.42 [0.23 to 0.77]	0.004
**Delayed surgery due to medical complication; n (%)**	8 (6.8)	17 (11.8)	0.50 [0.14 to 1.27]	0.17

Data are presented as mean (SD) or median [IQR] or n (%). 95% Confidence Intervals (CIs) are provided for the effect size, which corresponds to the estimated difference in medians for continuous data and the Odds Ratio (OR) for categorical outcomes.

**Table 4:** Intraoperative management.

	Clinical Pathway N = 117	Control N = 144	Effect Size (95%CI)	*p*
**Orthopedic Anesthesiologist;** **n (%)**	47 (40.2)	38 (26.6)	1.85 [1.09 to 3.13]	0.02
**Type of anesthesia; n (%)**				
Spinal	109 (94)	136 (95.1)	0.8 [0.27 to 2.37]	0.68
General	7 (6)	7 (4.9)		
**Surgical drains; n (%)**	18(15.4)	39(27.1)	0.49 [0.26 to 0.91]	0.023
**Urinary catheters**	17 (14.5)	21 (14.6)	0.99 [0.49 to 1.9]	0.99
**Hypothermia prevention;** **n (%)**	62 (53.8)	69 (47)	1.26 [0.76 to 2.09]	0.35
**Spinal opioids; n (%)**	83 (76.1)	88 (66.7)	1.59 [0.90 to2.82]	0.10
**Intravenous opioids; n (%)**	25 (21.7)	52 (37.1)	0.47 [0.26 to 0.82]	0.008
**Benzodiazepine sedation;** **n (%)**	22(19)	42 (29)	0.55 [0.30 to 0.99]	0.047
**PONV prophylaxis; n (%)**	66 (56.4)	51 (35.4)	2.36 [1.41 to 3.89]	0.001
**Intraoperative fluid volume crystalloid (mL)**	622.97 ± 243.99 500 [500.0–750.0]	613.21 ± 287.67 500 [500.0–700.0]	0 [–50 to 100]	0.18
**Intraoperative fluid volume colloid (mL)**	394.44 ± 121.13; 475 [250.0–500.0]	373.33 ± 137.40 400 [250.0–500.0]	0 [0 to 0]	0.81
**RBC transfusion; n (%)**	11(9.4)	13 (9)	1.04 [0.45 to 2.42]	0.91
**RBC transfusion units (total)**	1.6 ± 0.69; 1 [2.0–2.0]	1.25 ± 0.45 1 [1.0–1.0]	0 [0 to 1]	0.20
**Tranexamic administration; n (%)**	20 (17.5)	4 (2.9)	7.44 [2.4 to 22.48]	0.001
**Intraoperative complications; n (%)**	28 (24.1)	64 (45.7)	0.37 [0.22 to 064]	0.001
**Intraoperative hypotension; n (%)**	23 (20)	51 (36.4)	0.43 [0.24 to 0.77]	0.004
**Administration of vasopressors; n (%)**	23 (20)	57 (40.7)	0.36 [0.20 to 0.64]	0.001
**Arrythmia; n (%)**	2 (1.7)	9 (6.4)	0.25 [0.05 to 1.2]	0.053

RBC: red blood cells. PONV: Postoperative nausea and vomiting. Data are presented as mean (SD) or median [IQR] or n (%). 95% Confidence Intervals (CIs) are provided for the effect size, which corresponds to the estimated difference in medians for continuous data and the Odds Ratio (OR) for categorical outcomes.

**Table 5:** Postoperative management and complications.

	Clinical Pathway N = 117	Control N = 144	Effect Size (95%CI)	*p*
**Postoperative care unit stay (h)**	2.68 ± 1.19; 2.4 [2.0, 3.0]	2.94 ± 1.70; 2.5 [2.0–3.1]	0 [–4 to 0]	0.16
**Standardized analgesic protocol; n (%)**	109 (93.2)	38 (26.4)	38 [16.94 to 85.25]	0.001
**Early mobilization; n (%)**	87 (74.4)	59 (41.3)	4.12 [2.42 to 7.28]	0.001
**Time to mobilization (hours)**	31.2 ± 29.3 21 [18.0–38.0]	37.14 ± 23 24 [22.0–47.0]	–5.7 [–8 to –3]	0.001
**Time to ambulation (hours)**	78.4 ± 62.4; 65 [39.0–115.0]	88.6 ± 51.7 72 [48.0–120.0]	–14 [–26 to –2]	0.028
**Early food and drink intake; n (%)**	102 (87.2)	91(63.6)	3.49 [1.86 to 6.56]	0.001
**Glucose control; n (%)**	96 (82.1)	140 (97.2)	0.13 [0.04 to 0.39]	0.001
**Blood transfusion; n (%)**	50 (42.7)	64 (44.4)	0.95 [0.58 to 1.56]	0.78
**Composite of 30-day mortality or major complications; n (%)**	17 (14.5)	37 (25.7)	0.49 [0.26 to 0.92]	0.02
**Postoperative delirium; n (%)**	34 (29.3)	63 (43.8)	0.52 [0.31 to 0.88]	0.015
**Wound infection, n (%)**	3 (2.5)	1 (0.69)	3.79 [0.39 to 36.9]	0.21
**Urinary tract infection; n (%)**	15 (12.8)	16 (11.1)	1.18 [0.56 to 2.51]	0.65
**Ileus; n (%)**	35 (29.9)	42 (29.2)	1.04 [0.61 to 1.79]	0.86
**Need for reoperation; n (%)**	7 (6)	5 (3.5)	2.56 [0.75 to 8.75]	0.33
**Readmission; n (%)**	10 (8.8)	8 (5.7)	2.04 [0.76 to 5.45]	0.33
**Length of hospital stay; (days)**	15.50 ± 11.26; 13 [10.0–17.0]	14.34 ± 9.6 12 [9.0–16.0]	1 [–1 to 2]	0.32
**30-day mortality; n (%)**	5 (4.3)	12(8.3)	0.49 [0.16 to 1.43]	0.18
**1-year mortality; n (%)**	24 (20.5)	28 (19.4)	1.17 [0.64 to 2.16]	0.83

Data are presented as mean (SD) or median [IQR] or n (%). 95% Confidence Intervals (CIs) are provided for the effect size, which corresponds to the estimated difference in medians for continuous data and the Odds Ratio (OR) for categorical outcomes.

## Data Availability

All relevant data are presented within the manuscript.
